# Effect of Methylphenidate on Resting-State Connectivity in Adolescents With a Disruptive Behavior Disorder: A Double-Blind Randomized Placebo-Controlled fMRI Study

**DOI:** 10.3389/fpsyt.2021.662652

**Published:** 2021-06-17

**Authors:** Louise Pape, Koen van Lith, Dick Veltman, Moran Cohn, Reshmi Marhe, Wim van den Brink, Theo Doreleijers, Arne Popma

**Affiliations:** ^1^Department of Child and Adolescent Psychiatry, Amsterdam University Medical Center (UMC), Vrije Universiteit Amsterdam, Amsterdam, Netherlands; ^2^Department of Psychiatry, Amsterdam University Medical Center (UMC), Vrije Universiteit Amsterdam, Amsterdam, Netherlands; ^3^Amsterdam Institute for Addiction Research, Amsterdam University Medical Center (UMC), University of Amsterdam, Amsterdam, Netherlands; ^4^Institute for Criminal Law & Criminology, Leiden University, Leiden, Netherlands

**Keywords:** methylphenidate, disrucptive behavior, resting state – fMRI, adolecent, pharmacological fMRI

## Abstract

Some studies suggest that methylphenidate (MPH) might be an effective treatment for antisocial and aggressive behavior in adolescence. However, little is known about the mechanism of action of MPH in adolescents with this kind of psychopathology. MPH is a dopamine and norepinephrine reuptake inhibitor and thus it is likely to affect dopaminergic mesocorticolimbic pathways. This is the first study to investigate the effect of MPH on resting-state connectivity of three mesolimbic seed regions with the rest of the brain in clinical referred male adolescents with a disruptive behavior disorder (DBD). Thirty-six male DBD adolescents and 31 male healthy controls (HCs) were included. DBD subjects were randomly allocated to a single dose of MPH (DBD-MPH, *n* = 20) or placebo (DBD-PCB, *n* = 16). Seed-based resting-state functional connectivity of the nucleus accumbens (NAcc), amygdala, and ventral tegmental area (VTA) with the rest of the brain was compared between groups. The NAcc seed showed increased connectivity in DBD-PCB compared to HC with the occipital cortex, posterior cingulate cortex (PCC), precuneus, and inferior parietal lobule (IPL) and increased connectivity in DBD-PCB compared to DBD-MPH with occipital cortex, IPL, and medial frontal gyrus. The amygdala seed showed increased connectivity in DBD-PCB compared to HC with the precuneus and PCC. The VTA seed showed increased connectivity in the DBD-MPH compared to the DBD-PCB group with a cluster in the postcentral gyrus and a cluster in the supplementary motor cortex/superior frontal gyrus. Both NAcc and amygdala seeds showed no connectivity differences in the DBD-MPH compared to the HC group, indicating that MPH normalizes the increased functional connectivity of mesolimbic seed regions with areas involved in moral decision making, visual processing, and attention.

## Introduction

Antisocial behavior in children and adolescents constitutes a huge problem for society. It can lead to serious damage to themselves and their environment as well as to substantial economic costs (1). Until now, the effectivity of programs targeting these behaviors is only modest (2) and therefore it is crucial to develop better interventions. Neuroimaging research has shown that several brain areas are affected in disruptive and antisocial behavior [see (3) for review] and these findings may aid the development of new treatments.

Persistent patterns of antisocial behavior in adolescents are clinically diagnosed as disruptive behavior disorders (DBDs). DBD comprises oppositional defiant disorder (ODD), characterized by a pattern of irritable mood, defiant behavior, and/or vindictiveness, and conduct disorder (CD), characterized by frequent violation of social rules and rights of others. DBD is common in adolescents [prevalence 5–6% (4)] and currently there are no registered pharmacological interventions for DBD. Generally, guidelines advise to prescribe methylphenidate (MPH) only in DBD patients with comorbid ADHD or antipsychotics only in DBD patients with severe aggression (5). While MPH is not recommended for the routine treatment of DBD, some clinical studies indicated that MPH might have benefits even in DBD patients without comorbid ADHD (6–8), especially in more severe patients (9). In one study, it was suggested that MPH can enhance the effectiveness of psychotherapeutic interventions in DBD patients (10).

MPH is a dopamine re-uptake inhibitor; therefore, dopaminergic interference in brain circuits reported to be aberrant in patients with DBD may underlie the effectiveness of MPH in DBD. Moreover, there is suggestive evidence for the influence of dopamine genes on the development of DBD (11–13), which fits the hypothesis that the dopamine system is a potential target for pharmacological interventions. Finally, a recent meta-analysis (14) of whole-brain fMRI studies has revealed that compared to healthy controls (HCs), patients with DBD show decreased activations in the anterior cingulate cortex (ACC), the medial frontal cortex (MFC), and the ventral striatum (VS), while region-of-interest analyses also revealed abnormal amygdala activations [e.g., (15, 16)], i.e., brain areas that are part of the mesolimbic fronto-striatal dopamine pathway. In this pathway, the ventral tegmental area (VTA) releases dopamine and projects *via* the medial forebrain bundle to the nucleus accumbens (NAcc) and to other limbic structures, including the septum, hippocampus, amygdala, orbitofrontal cortex (OFC), ACC, and mPFC (17). Previous studies have shown that the connectivity of amygdala and NAcc with other structures of the fronto-striatal dopamine pathway are aberrant during decision making and emotion processing in DBD (18–21). Reduced top-down control over these limbic regions are suggested to underlie dysfunctional decision making and emotion processing in DBD (14, 22).

Resting-state (RS)-fMRI is a powerful tool because of its task-free nature and it was recently advocated to study the effects of MPH on intrinsic brain activity in DBD patients (23). However, due to a large variety in analytic approaches, comparing existing findings of RS-fMRI studies in DBD populations is problematic. Several studies have found reduced connectivity in or with the default mode network, hypothesized to be important in self-referential and moral processing (24–27), but in one study, this reduction was only seen after correction for ADHD symptoms (26). Furthermore, in line with the hypothesized aberrations in the mesolimbic fronto-striatal dopamine pathway in DBD, Aghajani et al. (22) found abnormally increased connectivity of the amygdala with a collection of regulatory paralimbic brain regions along with posterior cingulate, sensory associative, and striatal regions in CD youth high on CU (callous-unemotional) traits.

Literature on the effect of MPH on resting-state connectivity is beginning to emerge, with one study in patients with a cocaine use disorder showing an effect on the connectivity within the mesolimbic fronto-striatal dopamine pathway (28) and another study in HCs showing reduced NAcc connectivity with other parts of the reward circuit (29).

In the current between-subject, randomized double-blind placebo-controlled pharmacologic fMRI study, we investigated for the first time the effect of MPH on mesolimbic pathways in adolescent males with DBD using a seed-based approach that was used before by Konova et al. (28). We hypothesized that (1) male adolescent DBD patients compared to HCs show an abnormal resting-state connectivity pattern of the subcortical NAcc, amygdala, and VTA seeds with cortical areas involved in emotion and reward processing and (2) MPH will normalize this abnormal connectivity pattern within the mesolimbic pathways in male adolescent DBD patients.

## Materials and Methods

The present study is part of a larger project, approved by the Central Committee on Research Involving Human Subjects (CCMO) and registered in the Dutch Trial Register (NTR, www.trialregister.nl; number NTR 4088). All participants and their legal guardian(s) signed informed consent prior to participation.

Participants and their parents or custodians were visited at home for a structured psychiatric interview and questionnaires. On a second occasion, participants visited the Spinoza Center for neuroimaging in Amsterdam (The Netherlands) for MRI scanning. Participants received a financial remuneration of €100 in vouchers.

### Participants

Participants included adolescent DBD patients and HCs. Patients were 14- to 17-year-old males, diagnosed with either ODD, CD, or both, recruited at “De Bascule,” an academic center for child and adolescent psychiatry in Amsterdam. In order to match the HCs with the DBD participants, HCs were recruited *via* regular secondary schools in neighborhoods with a low/middle socioeconomic status (SES) and lower/mean education level in the greater Amsterdam region. At the start of the study, patients with DBD (*n* = 57) were randomly allocated to one of three groups: one group received MPH (DBD-MPH; *n* = 25), one group received an identical placebo (DBD-PCB; *n* = 24), and one group did not receive any intervention (*n* = 8). Due to low inclusion rates, we decided to stop assigning patients with DBD to the non-intervention group and excluded them from analyses. Overall, 88 male adolescents were included, 49 patients with a clinical DBD diagnosis and 39 age/SES/education-matched HCs. General exclusion criteria were as follows: (1) violation of MRI safety criteria, (2) use of psychotropic medication other than MPH or dextroamphetamine or not willing or able to refrain from MPH or dextroamphetamine use 72 h prior to scanning, (3) IQ below 80, (4) actual steroid use, (5) history of head trauma, (6) neurological disorder, (7) current or lifetime history of psychosis, (8) Tourette's syndrome, and (9) pervasive developmental disorder. Furthermore, HCs were excluded if they had a history of antisocial behavior (e.g., police contact, expelled from school) or a current psychiatric diagnosis.

### Assessment

The parent and youth versions of the Diagnostic Interview Schedule for Children (DISC-IV) (30) were used to assess the clinical diagnosis of ODD and CD, as well as ADHD. To estimate IQ (31), the vocabulary and block design subtests of the Wechsler intelligence scale for children (WISC) were used (32). Further exclusion based on the criteria described above was applied. Patients and HCs were matched on age, SES, and education. SES of each participant was based on data from The Netherlands Institute for Social Research (33). For each zip code, a standardized score was provided, i.e., −1 (low), 0 (intermediate), or +1 (high). The Dutch version of the Youth Psychopathy Inventory (YPI) (34)^1^ was used to assess CU traits.

### Methylphenidate

In this double-blind study, DBD patients were randomly assigned to either a single dose of 0.3–0.4 mg/kg MPH (DBD-MPH) or a single dose of placebo (Albocin) (DBD-PCB) ~2 h before the start of the resting-state scan. This dosage was similar to previous studies in patients with ADHD (35, 36). Given the previously reported correlation of resting-state connectivity and CU traits in DBD (37), randomization was stratified for a high vs. a low CU score on the YPI with a CU cutoff score of 27 (38). Healthy controls did not receive any type of pharmacological intervention.

### Image Acquisition

Resting-state fMRI images were acquired on a 3-T whole-body MR scanner (Philips Achieva XT, Best, The Netherlands) using a 32-channel head coil with a gradient-echo EPI sequence (TR = 2,000 ms; TE = 27.63 ms; flip angle = 76.1°; FOV = 240 × 240 mm^2^; acquisition matrix size = 80 × 80; 37 3-mm slices with a 0.3-mm gap; 4 dummy scans, 200 volumes total). Participants were instructed to fixate on a crosshair presented on the middle of a screen and to relax. In addition, 3D T1-weighted anatomical images were acquired using an axial sequence (TR = 8.2 ms; TE = 3.8 ms; flip angle = 8°; 220 slices; resolution = 1 × 1 × 1 mm^3^).

#### Data Pre-processing

Anatomical images were skull-stripped, segmented, and normalized to the MNI152 template using the Advanced Normalization Tools (ANTs) (39). Standard preprocessing steps were performed and included the following: motion-correction, registration to the anatomical brain, intensity normalization, nuisance signal removal [including 24 motion parameters, five principal components derived from the CSF and white matter signals [according to the compcor method, see Behzadi et al. (40)], the average signal from the CSF, as well as the linear and quadratic trends], and temporal filtering between 0.01 and 0.1 Hz.

**Figure 1:**
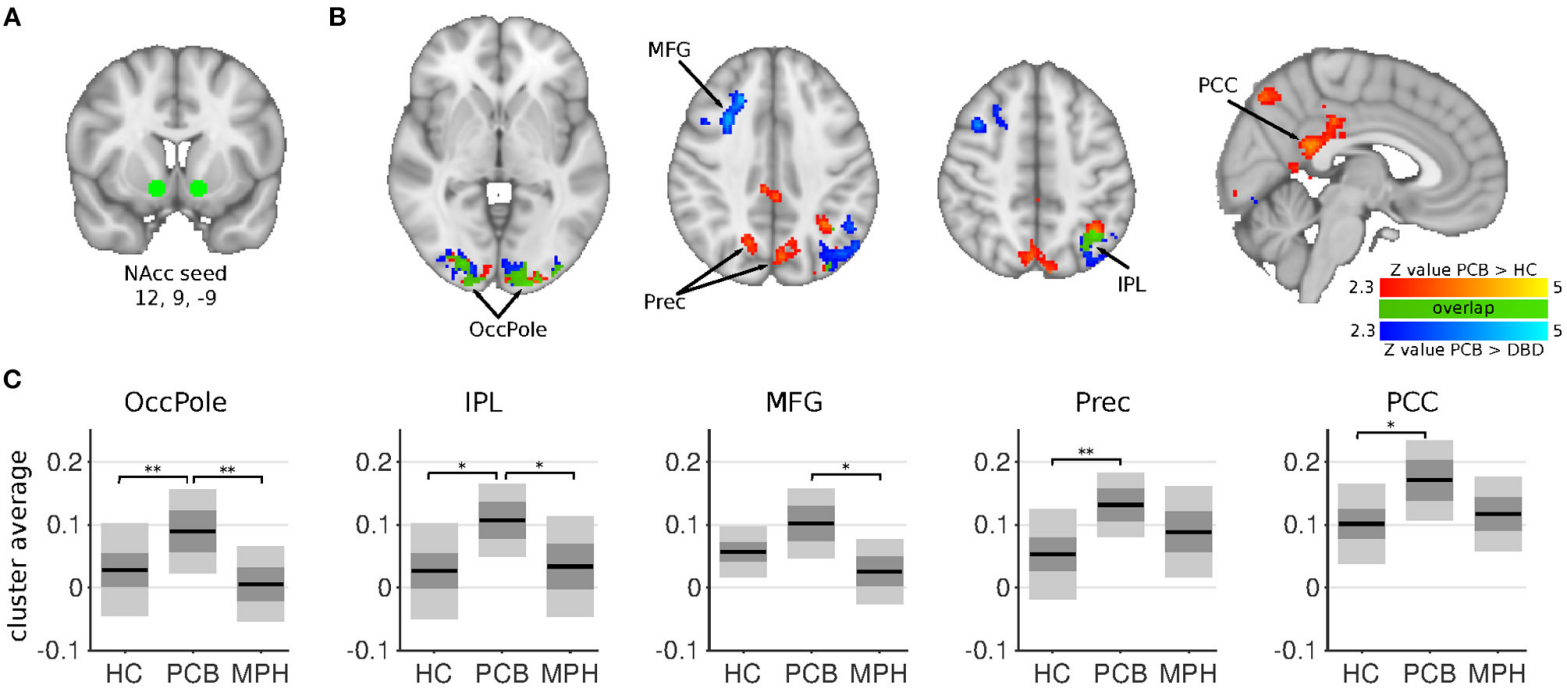
NAcc connectivity differences between HC, DBD-PCB, and DBD-MPH groups. **(A)** Location of the NAcc seed. **(B)** Clusters showing significant connectivity differences (*Z* > 2.3, cluster *p* < 0.05). **(C)** Average Fisher-*Z* transformed NAcc connectivity in the significant clusters. Dark gray patches represent the 95% confidence interval and light gray patches represent the standard deviations. Black lines represent the group means. All shown coordinates are in MNI space. NAcc, nucleus accumbens; OccPole, Occipital pole; Prec, Precuneus; MFG, medial frontal gyrus; IPL, inferior parietal lobule; PCC, posterior cingulate cortex; HC, healthy control; PCB, placebo; MPH, methylphenidate; *cluster-*p* < 0.05; **cluster *p* < 0.001.

**Figure 2:**
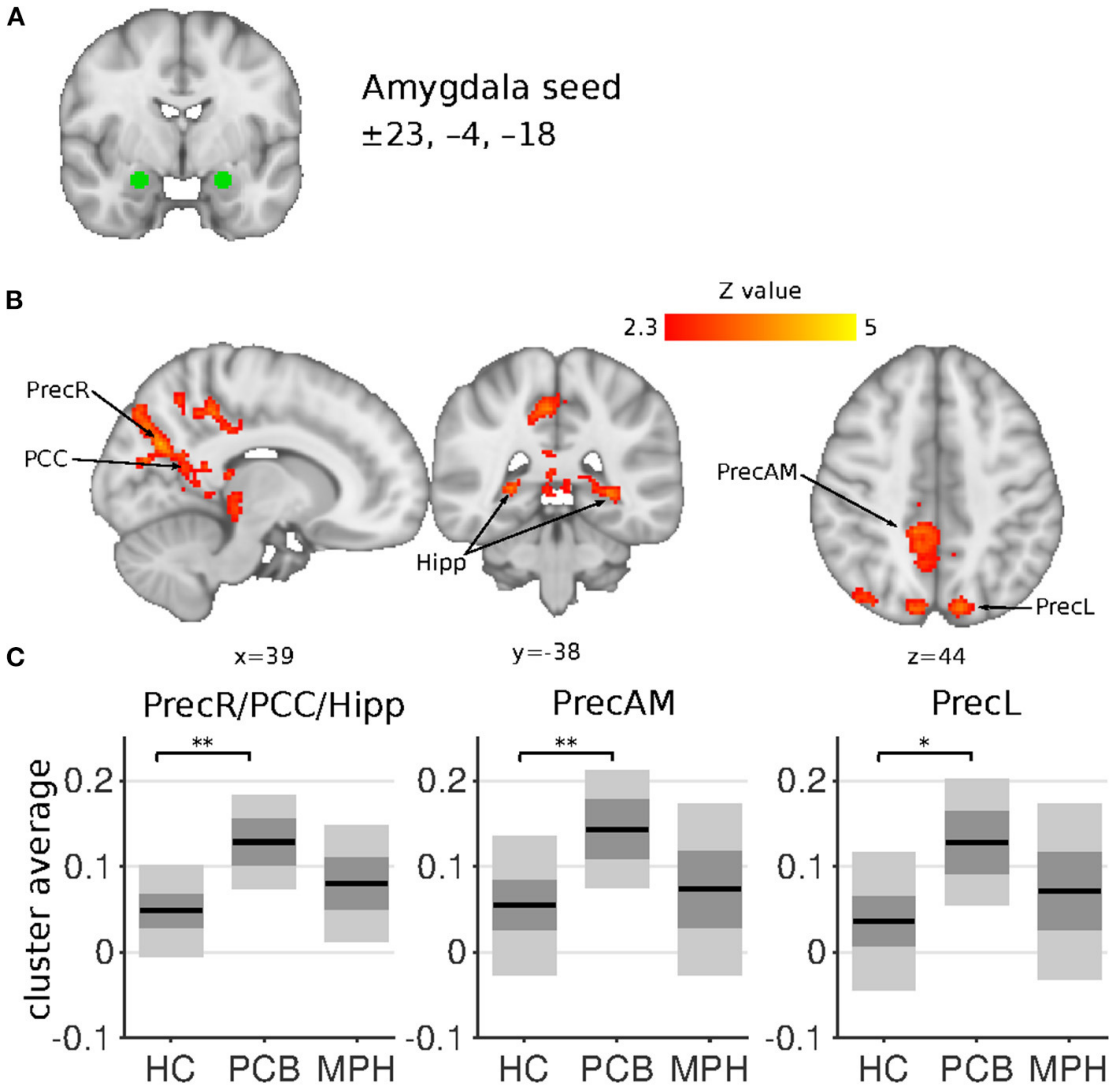
Amygdala connectivity differences between HC, DBD-PCB, and DBD-MPH groups. **(A)** Location of the amygdala seed. **(B)** Clusters showing significant connectivity differences (*Z* > 2.3, cluster *p* < 0.05). **(C)** Average Fisher-*Z* transformed amygdala connectivity in the significant clusters. Dark gray patches represent the 95% confidence interval and light gray patches represent the standard deviations. Lines represent the group means. All shown coordinates are in MNI space. PrecR, right posterior precuneus; PCC, posterior cingulate cortex; Hipp, hippocampus; PrecAM, medial anterior precuneus; PrecL, left posterior precuneus; HC, healthy control; PCB, placebo; MPH, methylphenidate; *cluster-*p* < 0.05; **cluster *p* < 0.001.

**Figure 3:**
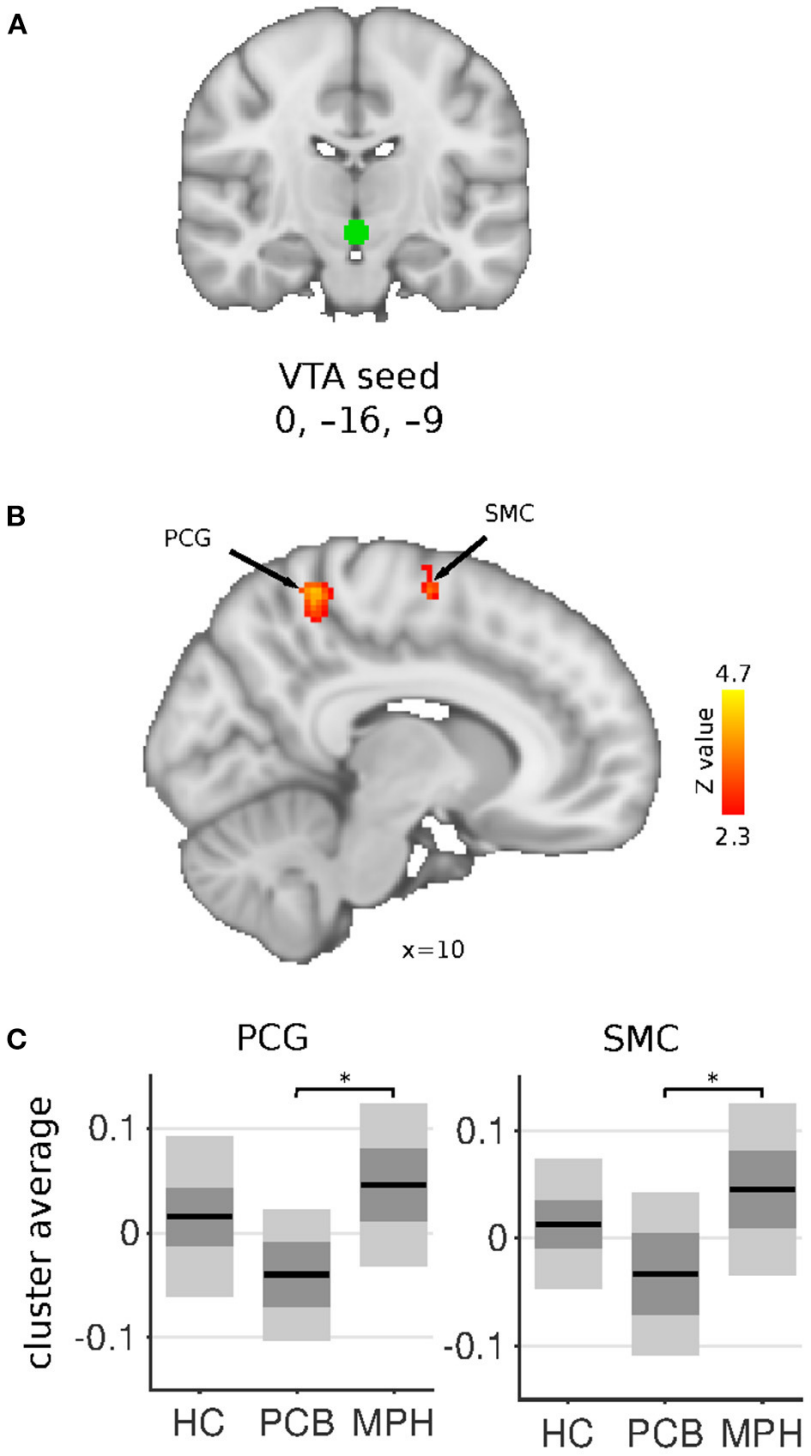
VTA connectivity differences between HC, DBD-PCB, and DBD-MPH groups. **(A)** Location of the VTA seed. **(B)** Clusters showing significant connectivity differences (*Z* > 2.3, cluster *p* < 0.05). **(C)** Average Fisher-Z transformed VTA connectivity in the significant clusters. Dark gray patches represent the 95% confidence interval and light gray patches represent the standard deviations. Lines represent the group means. All shown coordinates are in MNI space. VTA, ventral tegmental area; PCG, post-central gyrus; SMC, supplementary motor cortex; HC, healthy controls; PCB, placebo; MPH, methylphenidate; *cluster-*p* < 0.05.

Data preprocessing was performed using an alpha version of the Configurable Pipeline for the Analysis of Connectomes (C-PAC version 0.3.9, http://fcp-indi.github.io./). Three seed regions (NAcc, amygdala, and VTA) were defined by centering bilateral spheres with a radius of 5 mm at the coordinates shown in Figures 1–3. The location of these seeds was based on the study by Konova et al. (28). To reduce multiple testing issues, we decided not to include the hippocampus, thalamus, and rostral ACC as seed regions, because these areas are not of primary interest in DBD populations. Time series from these seeds were extracted by warping the preprocessed resting-state images to MNI space and averaging the signal in the seed regions. To test our hypotheses, whole-brain correlation maps were calculated separately for each bilateral seed region by correlating the time series from each voxel (in MNI space) with the seed time course. Subsequently, correlation maps were smoothed by applying a 4-mm FWHM Gaussian filter and transformed using Fisher R-to-Z to improve normality.

### Statistical Analysis

Eventual group differences in age, IQ, SES, head motion, tobacco, alcohol, and cannabis use were analyzed with ANOVAs. Whole-brain voxel-wise group analyses were performed using the Local Analysis of Mixed Effects (FLAME) with the FLAME1+2 option from the FMRIB Software Library (FSL, http://fsl.fmrib.ox.ac.uk) for each seed (VTA, NAcc, Amygdala) separately. Age and IQ were included as covariates in the model. Multiple comparisons were corrected for by using Gaussian Random Field theory implemented in the FSL script *easythresh* (*Z* > 2.3; cluster significance *p* < 0.05 corrected).

A study-specific mask was generated by only including voxels where 50% of the subjects had non-zero values. Planned comparisons between groups (DBD-PCB vs. DBD-MPH, DBD-PCB vs. HC) were performed using *t*-tests. To investigate the possible confounding effect of ADHD, we conducted whole-brain voxel-wise regression analyses for each seed in the DBD-PCB group only, with the number of ADHD symptoms as independent variable. We implemented this approach because ADHD symptoms were collinear with group status and could therefore not be added as an independent variable to the second-level model. We checked for overlap in brain areas significantly related to ADHD symptoms and clinical group status. Additionally, to further investigate the relationship between functional connectivity and ADHD symptoms, we conducted Pearson correlation analyses between average connectivity values of the significant clusters and number of ADHD symptoms. Furthermore, average Fisher-*Z* transformed correlation values from significant clusters were extracted to examine possible associations with reported number of days with cannabis use in the prior 30 days using correlation analysis.

## Results

### Subject Characteristics

Out of the 88 included subjects, 85 completed the resting-state scan (36 HC and 49 DBD). Of these, 18 were excluded (5 HC and 13 DBD): 2 HC because they met diagnostic criteria for ODD and/or ADHD and 4 DBD because they did not meet DBD criteria on the DISC-IV, 1 HC and 1 DBD because of an IQ <80, and 2 HC, 5 DBD-PCB, and 3 DBD-MPH subjects because of excessive head motion based on their average motion statistic [mean framewise displacement calculated according to the Jenkinson method (41)]. The final analyses thus included 31 HC and 36 DBD subjects, 16 in the placebo group and 20 in the MPH group. Groups differed in tobacco and cannabis use. *Post-hoc* testing revealed that both DBD groups used more tobacco compared to the HC group and that the DBD-MPH group used more cannabis compared to the HC and the DBD-PCB groups. There was no difference in the history of stimulant medication use between the DBD groups. There were no significant differences in age, IQ, mean motion, or SES between the three groups (see Table 1 for statistics).

**Table 1:** Characteristics of the HC and DBD groups.

	**HC**	**MPH**	**PCB**	**Group differences**
	***n* = 31**	***n* = 20**	***n* = 16**	
Age, mean years (SD)	15.9 (1.1)	16,1 (1.0)	15.8 (0.9)	*F*_2, 64_ = 0.31, *p* > 0.74
IQ, mean (SD)	98.2 (11.1)	95.2 (9.7)	95.8 (10.8)	*F*_2, 64_ = 0.61, *p* > 0.55
SES, mean (SD)	0.2 (1.1)	0.0 (1.6)	−0.3 (1.3)	*F*_2, 62_ = 0.92, *p* > 0.40
Right handed (%)	18 (86%)	19 (95%)	16 (100%)	Fisher's exact *p* = 0.55
Motion (FD Jenkinson)	0.15 (0.058)	0.17 (0.087)	0.15 (0.063)	*F*_2, 64_ = 0.37, *p* > 0.69
Low/high CallousUnemotional		3/17	3/13	Fisher's exact *p* > 0.95
ADHD (%)	0 (0%)	7 (35%)	10 (63%)	Fisher's exact *p* = 0.18^a^
ODD (%)	0 (0%)	5 (25%)	6 (38%)	Fisher's exact *p* = 0.48^a^
CD (%)	0 (0%)	7 (35%)	4 (25%)	Fisher's exact *p* = 0.71^a^
CD & ODD (%)	0 (0%)	8 (40%)	6 (38%)	Fisher's exact *p* > 0.99^a^
Lifetime stimulant use	0 (0%)	10 (50%)	7 (44%)	Fisher's exact, *p* = 0.75^a^
Stimulant use last week (%)	0 (0%)	4 (20%)	4 (25%)	Fisher's exact *p* > 0.99
MPH study dose (mg), mean (SD)		22.5 (2.7)		
Tobacco use, mean (SD), cigarettes/day	0.7 (3.5)	7.1 (5.9)	4.4 (6.6)	*F*_2, 62_ = 9.48 *p* = 0.00^b^
Alcohol use, mean (SD), days/month	0.90 (2.35)	2.5 (3.4)	1.1 (1.5)	*F*_2, 60_ = 2.33 *p* = 0.11
Cannabis use, mean (SD), days/month	0.1 (0.4)	11.9 (12.2)	2.6 (5.5)	*F*_2, 60_ = 16.32 *p* = 0.00^b^^,^^c^

a *Comparison between DBD-MPH and DBD-Placebo group*.

b *Significant difference between HC and total DBD group*.

c *Significant difference between DBD-PCB and DBD-MPH*.

*ADHD, Attention Deficit/Hyperactivity Disorder; DBD, Disruptive Behavior Disorder; FD, Framewise displacement, calculated according to the Jenkinson method (41); HC, Healthy Control; IQ, Intelligence Quotient; MPH, Methylphenidate; ODD, Oppositional Defiant Disorder; PCB, Placebo; SD, Standard Deviation; SES, Socioeconomic Status*.

### Connectivity Differences

The NAcc seed showed increased connectivity in the DBD-PCB compared to the HC group with five clusters (Figure 1): two clusters in the bilateral occipital cortex (BA 18, primary visual), a cluster in the posterior cingulate cortex (PCC), a cluster in the precuneus, and a cluster in the left inferior parietal lobule (IPL). The NAcc seed also showed increased connectivity in the DBD-PCB compared to the DBD-MPH group with four clusters: two clusters in the bilateral occipital cortex, a cluster in the left IPL, and a right medial frontal gyrus (BA 8/44/47). The amygdala seed showed increased connectivity in the DBD-PCB compared to the HC group (Figure 2) with a cluster comprising parts of the right posterior precuneus, PCC, and hippocampus, a cluster in the left posterior precuneus, and a cluster in the anterior medial precuneus.

Both the NAcc and amygdala seeds showed no connectivity differences in the DBD-MPH compared to the HC group.

The VTA seed showed increased connectivity in the DBD-MPH compared to the DBD-PCB group with a cluster in the postcentral gyrus and a cluster in the supplementary motor cortex/superior frontal gyrus (Figure 3). See Table 2 for a summary of the statistics.

**Table 2:** Clusters and coordinates of between group differences in connectivity.

				**Peak voxel MNI Coordinates**		
**Region(s)**	***N* voxels**	***p*-value**	**MAX *Z*-value**	***x***	***y***	***z***
**NAcc seed**						
*PCB > HC*						
Precuneus Bilateral	1,100	1.28e−05	4.55	−12	−66	28
Occipital pole L	757	0.000408	4.61	−26	−98	6
Posterior cingulate cortex	495	0.00833	3.98	6	−30	36
Occipital pole R	470	0.0114	3.86	24	−98	10
Inferior parietal lobule L	434	0.018	4.01	−36	−58	44
*PCB > MPH*						
Occipital pole L	1123	3.41e−05	4.36	−24	−98	10
Inferior parietal lobule L	750	0.00107	3.9	−52	−66	40
Occipital pole R	734	0.00125	3.84	14	−100	13
Medial frontal gyrus R	508	0.0136	4.71	30	16	40
**Amygdala seed**						
*PCB > HC*						
Posterior precuneus R/ Posterior cingulate cortex/ Hippocampus Bilateral	1,904	9.03e−09	4.24	12	−68	28
Anterior precuneus Medial	830	0.000158	3.91	6	−42	48
Posterior precuneus L	465	0.0109	3.57	−12	−80	44
**VTA seed**						
*MPH > PCB*						
Supplementary motor cortex/Superior frontal gyrus R	394	0.00469	3.83	16	−4	62
Postcentral gyrus R	265	0.0488	4.75	8	−46	62

*All Z-values are corrected for multiple comparisons at the cluster-level (Z > 2.3; p <0.05). NAcc, nucleus accumbens; VTA, ventral tegmental area; L, left; R, right*.

### Effect of Comorbid ADHD

**Figure 4:**
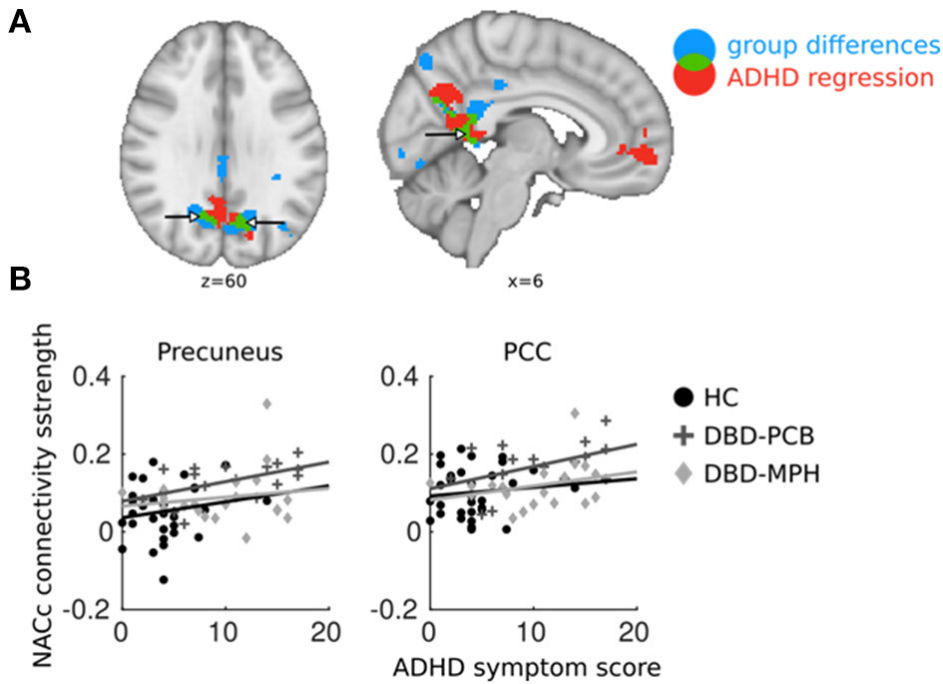
Overlapping regions between ADHD regression and DBD group comparisons. ADHD regression analyses were performed in DBD subjects only. **(A)** In blue, brain areas of NAcc connectivity significantly different between DBD-PCB and HC groups; in red, NAcc connectivity significantly related to ADHD symptoms; in green, overlap between the two. **(B)** Average connectivity strength in PCC and precuneus plotted against ADHD symptom scores (to avoid double-dipping, we used the cluster-averages from the DBD-PCB vs. HC comparison). ADHD, Attention deficit/hyperactivity disorder; PCC, Posterior Cingulate Cortex; NAcc, Nucleus Accumbens; HC, Healthy control; PCB, Placebo; MPH, Methylphenidate. *x* and *z* coordinates are in MNI space.

Results from the regression analysis in the DBD-PCB group only showed a positive relation between ADHD symptoms and NAcc connectivity with the PCC and precuneus. This cluster was partially overlapping with the clusters of increased connectivity in the DBD-PCB vs. the HC group (Figure 4A). The other seeds did not show any overlapping regions of clinical group and ADHD-related connectivity differences. Average connectivity strength between the NAcc seed and PCC and precuneus were plotted against ADHD symptom scores (to avoid double-dipping, we used the cluster-averages from the DBD-PCB vs. HC comparison). These scatter plots are purely explorative, but they showed that the normalizing effect of MPH was independent of the number of ADHD symptoms (Figure 4B). Correlation analyses showed that these correlations were significant in the DBD-PCB group between ADHD symptoms and NAcc–precuneus connectivity (*r* = 0.51, *p* = 0.04) and at-trend to significant between ADHD symptoms and NAcc–PCC connectivity (*r* = 0.46, *p* = 0.07). Within the DBD-MPH group and the HC group, there were no significant correlations between ADHD symptoms and NAcc–precuneus connectivity (HC, *r* = 0.17, *p* = 0.34; DBD-MPH, *r* = 0.15, *p* = 0.53) and ADHD symptoms and NAcc–PCC connectivity (HC, *r* = 0.11, *p* = 0.57; DBD-MPH, *r* = 0.29, *p* = 0.21).

### Effect of Cannabis Use

We found no significant correlations between the reported number of days with cannabis use in the prior 30 days and values within the significant clusters (see Supplementary Table 1).

## Discussion

This is the first study investigating the effect of a single dose of MPH on resting-state functional connectivity of NAcc, amygdala, and VTA-centered networks in male adolescents diagnosed with a DBD. We replicated previous findings on the dysfunction of amygdala- and NAcc-centered networks involved in reward, decision making, empathy, and attention. More importantly, this study is the first to suggest that MPH normalizes these aberrant networks in male adolescents with DBD.

Comparing DBD patients in the placebo condition (DBD-PCB) with matched HCs revealed the presence of hyperconnectivity of the amygdala with the precuneus, PCC, and left intra-parietal lobule (IPL). In addition, we found hyperconnectivity of the NAcc with the occipital pole, PCC, the precuneus, and the left IPL in DBD patients compared to HCs. These results are in line with, and extend, recent studies by Aghajani et al. (22) and DeWitt et al. (42), which also showed increased connectivity of the amygdala with precuneus/PCC/IPL in adolescents with CD and risk-taking behaviors, respectively. Other studies in DBD patients have also reported resting-state abnormalities in the precuneus, PCC, and IPL (43, 44). PCC, precuneus, and IPL are all key regions in the default mode network (DMN), mainly active during rest and involved in self-referential activity and moral decision making (24). Previous studies have shown decreased connectivity of the amygdala with these DMN-subregions during passive avoidance learning, emotional faces viewing and moral judgements in DBD patients (15, 19, 21). Although directly comparing resting-state and task-related functional connectivity should be done with great caution, we may speculate that increased connectivity of the amygdala and NAcc with the DMN could hinder other networks to successfully “recruit” the amygdala and NAcc. This might lead to impaired emotion processing, reward processing, and learning, which have all been implicated in patients with DBD (3). Alternatively, since the DMN is primarily active during rest, increased connectivity with amygdala and NAcc suggests activation of these structures during rest. This increased “interference” of emotional and motivational input to the DMN may disrupt normal functioning of the DMN, leading to impaired moral reasoning and social judgement.

We also found increased connectivity of NAcc with the occipital pole. Previous resting-state studies, although using different metrics or network approaches, reported aberrances in similar occipital regions (43, 45, 46). A recent study related increased connectivity of the VS with the occipital pole with high reward sensitivity, suggesting that VS modulates visual attention to rewards (47). The hyperconnectivity of NAcc with the occipital pole might reflect impairments in attention reported in DBD (48) and antisocial adults (49) while Lu et al. (44) interpreted this finding as reflecting aberrant top-down control. Furthermore, increased NAcc–occipital connectivity may indicate heightened reward motivation since previous research showed increased visual cortex activation and visual stimulus processing during states of reward motivation (50–54).

In the current study, we did not find connectivity abnormalities between any of the seeds and other regions of the mesolimbic fronto-striatal dopamine pathway, whereas the meta-analyses of Alegria et al. (14) revealed functional aberrances in (among other regions) the ACC, the MFC, and the VS. Therefore, we *post-hoc* tested group differences in connectivity between the seeds and a ROI containing the Medial Frontal Orbital cortex, Rectus, and the anterior and mid Cingulum. In line with the results of the planned analyses, the *post-hoc* testing also revealed no significant group differences in functional connectivity between the seeds and the created ROI. As stated before, the lack of significant differences may have been due to technical issues (low vmPFC signal). However, the previously reported resting-state connectivity abnormalities between the amygdala and the mPFC and ACC (22) and task-related connectivity abnormalities between amygdala and vmPFC (15, 18) and ACC (16) were observed in patients high on CU traits and therefore may reflect CU traits rather than DBD. A previous study of our own group likewise indicated that CU traits have unique resting-state correlations (37).

The comparison of DBD-PCB and DBD-MPH provides evidence for a normalizing effect of MPH on aberrant resting-state connectivity between the NAcc and the occipital cortex and the IPL. Normalization of NAcc-PCC/Precuneus connectivity was suggested by the cluster-averages plots in Figure 1, but this effect was not statistically significant. We did not observe differences between DBD-MPH and HC regarding NAcc or amygdala connectivity, providing some further, albeit indirect, evidence for MPH-associated normalization.

The VTA, which is the source of the mesolimbic fronto-striatal dopamine pathway, showed increased connectivity in sensorimotor areas in DBD-MPH vs. DBD-PCB. However, no differences were found between DBD-PCB and HC groups, suggesting a pharmacological effect within the study population.

Our findings indicate potential mechanisms underlying the previously demonstrated positive effect of MPH on DBD symptoms (6–10). For example, one may speculate that the previous reported positive effect on DBD symptoms is due to a direct normalizing effect on reward sensitivity, decision making, and empathy, all found to be disturbed in DBD (3). Although highly speculative, MPH may also have an indirect effect on clinically relevant behavior by normalizing (visual) attention. Since we cannot relate the hyperconnectivity of the NAcc with the occipital lobe to increased or decreased attention, there are two possible explanations for the relation between attentional problems and DBD symptoms. Visual attention is important in focusing on relevant information while ignoring irrelevant information (55). A hyperfocus on information relevant for obtaining a goal leads to rigid behavior due to difficulties in adjusting behaviors (56). Alternatively, lowered attention may result in problems in self-regulation in situation with distracting information (49). Our results showed a significant decrease in limbic–prefrontal connectivity, suggesting that MPH may modulate top-down control of motivation and affect (14). Alternatively, the normalizing effects of MPH might be due to increased capacities to switch attention to relevant stimuli (57). Whether MPH indeed normalizes attention in DBD should be studied in studies measuring visual attention in DBD during a MPH challenge and/or after a MPH treatment. Overall, normalizing attention might increase the susceptibility of DBD patients for positive parenting or psychotherapy.

Analyses to explore the possible confounding effect of comorbid ADHD indicated that the observed hyperconnectivity of the NAcc with the PCC and precuneus in DBD-PCB vs. HC was partially overlapping with hyperconnectivity related to ADHD symptoms in the DBD-PCB group. As such, the increased NAcc–DMN connectivity in DBD may also reflect ADHD pathology. However, the normalizing effect of MPH on NAcc–DMN connectivity was independent of the number of ADHD symptoms.

This study has several strengths and limitations. The first strength is that we included a clinical sample representing common but severe patients and a sample of HCs matched on age, IQ, and SES. Second, we had a double-blinded, placebo-controlled design. Third, we had a hypothesis-driven approach to analyze the data and explored the potential effects of ADHD. Fourth, patients with DBD in the MPH condition received a clinical dosage MPH, based on their body weight.

There are also some limitations. Due to the use of a between-subject design, although we used pre-stratification on potential confounders (e.g., CU traits) and did *post-hoc* correction for medication and substance use, pre-existing connectivity differences between the DBD groups cannot be excluded. Future research should try to use a double-blind crossover design, although this approach is even more challenging due to the characteristics of patients with DBD (23). For ethical reasons, the HCs did not receive a (placebo) intervention so we cannot fully exclude that the differences between DBD-PCB and HCs are due to a placebo effect. Since our final DBD groups were relatively small, the results should be replicated in larger samples. Larger samples are also needed to clarify the potential effect of cannabis use on resting-state connectivity (see Supplementary Table 1). While our seed-based analyses were hypothesis driven and designed to study the influence of MPH on resting-state connectivity in patients with DBD, the aberrances seen in DBD-PCB are difficult to compare with previous resting-state research in patients with DBD. Since there is heterogeneity in instructions (eyes open or closed during scanning), analysis method and patient selection (e.g., excluding comorbid ADHD, community, clinical, or incarcerated samples) replication of our DBD-PCB findings are needed. To increase the usefulness of resting state in antisocial populations, future researchers should use standardized methods. We suggest future researchers to instruct their participants to keep their eyes open with fixation on a cross since this induces greater reliability in, e.g., default mode and attentional networks (58), to use a seed-based approach focusing connections in networks involved in reward and emotion processing, and to use standard correction for ADHD symptoms.

In conclusion, we replicated previous findings of hyperconnectivity of limbic areas and networks involved in reward and emotion processing and in attention in patients with DBD and showed that a single dose of MPH normalizes this hyperconnectivity in DBD patients.

## Data Availability Statement

The raw data supporting the conclusions of this article will be made available by the authors, without undue reservation.

## Ethics Statement

The studies involving human participants were reviewed and approved by Central Committee on Research Involving Human Subjects (CCMO), The Netherlands. Written informed consent to participate in this study was provided by the participants' legal guardian/next of kin.

## Author Contributions

LP: design, data acquisition, data analysis, drafting, and revision. KL: conception, design, data acquisition, drafting, and revision. DV and WB: conception, design, revision, and final approval. MC: conception, design, and revision. RM: revision and final approval. TD: conception, design, and final approval. AP: conception, design, revision, final approval, and accountability. All authors contributed to the article and approved the submitted version.

## Conflict of Interest

The authors declare that the research was conducted in the absence of any commercial or financial relationships that could be construed as a potential conflict of interest.

## Footnotes

^1^Das J, de Ruiter C. *Youth Psychopathic Traits Inventory: Geautoriseerde Nederlandse Vertaling [Youth Psychopathic Traits Inventory; Authorized Dutch Translation]*. (2003).
